# Monoclonal Antibody AP3 Binds Galactomannan Antigens Displayed by the Pathogens *Aspergillus flavus, A. fumigatus*, and *A. parasiticus*

**DOI:** 10.3389/fcimb.2019.00234

**Published:** 2019-07-16

**Authors:** Max Schubert, Sheng Xue, Frank Ebel, Annegret Vaggelas, Vadim B. Krylov, Nikolay E. Nifantiev, Ivana Chudobová, Stefan Schillberg, Greta Nölke

**Affiliations:** ^1^Department of Plant Biotechnology, Fraunhofer Institute for Molecular Biology and Applied Ecology IME, Aachen, Germany; ^2^Institute for Translational Medicine, College of Medicine, Qingdao University, Qingdao, China; ^3^Faculty of Veterinary Medicine, Institute for Infectious Diseases and Zoonoses, Ludwig-Maximilians-University Munich, Munich, Germany; ^4^N.D. Zelinsky Institute of Organic Chemistry, Russian Academy of Sciences, Moscow, Russia; ^5^Institute for Phytopathology, Justus Liebig University Giessen, Giessen, Germany

**Keywords:** *Aspergillus* antigen, detection assay, epitope identification, galactofuranose, glycobiology

## Abstract

*Aspergillus fumigatus* and *A. flavus* are the fungal pathogens responsible for most cases of invasive aspergillosis (IA). Early detection of the circulating antigen galactomannan (GM) in serum allows the prompt application of effective antifungal therapy, thus improving the survival rate of IA patients. However, the use of monoclonal antibodies (mAbs) for the diagnosis of IA is often associated with false positives due to cross-reaction with bacterial polysaccharides. More specific antibodies are therefore needed. Here we describe the characterization of the *Aspergillus*-specific mAb AP3 (IgG1κ), including the precise identification of its corresponding antigen. The antibody was generated using *A. parasiticus* cell wall fragments and was shown to bind several *Aspergillus* species. Immunofluorescence microscopy revealed that AP3 binds a cell wall antigen, but immunoprecipitation and enzyme-linked immunosorbent assays showed that the antigen is also secreted into the culture medium. The inability of AP3 to bind the *A. fumigatus* galactofuranose (Gal*f* )-deficient mutant Δ*glfA* confirmed that Gal*f* residues are part of the epitope. Several lines of evidence strongly indicated that AP3 recognizes the Gal*f* residues of *O*-linked glycans on *Aspergillus* proteins. Glycoarray analysis revealed that AP3 recognizes oligo-[β-D-Gal*f*-1,5] sequences containing four or more residues with longer chains more efficiently. We also showed that AP3 captures GM in serum, suggesting it may be useful as a diagnostic tool for patients with IA.

## Introduction

The genus *Aspergillus* comprises 339 filamentous fungi that are ubiquitous in nature and have many potential applications in biotechnology, but some species also pose a risk to human and animal health (Samson et al., 2014). *A. niger* and *A. oryzae* are widely used for fermentation in the food industry and for the production of hydrolytic enzymes (Biesebeke and Record, 2008). In contrast, *A. fumigatus* and *A. flavus* are major pathogens responsible for allergic bronchopulmonary aspergillosis (ABPA), chronic pulmonary aspergillosis (CPA) and invasive aspergillosis (IA), which can be fatal in immunocompromised patients, such as carriers of human immunodeficiency virus, or patients receiving transplants of allogeneic stem cells or solid organs (Singh and Paterson, 2005; Krishnan et al., 2009). The early detection of biomarkers elicited by invasive *Aspergillus* species is necessary to achieve effective antifungal therapy outcomes (Hedayati et al., 2007; Walsh et al., 2008).

*A. flavus* is responsible for 15–20% of reported IA cases (Perfect et al., 2001; Krishnan et al., 2009). Furthermore, *A. flavus* and *A. parasiticus* also infect plants, where they produce highly carcinogenic secondary metabolites known as aflatoxins, particularly when they grow on oil-rich staple crops under field and storage conditions (Villers, 2014). These aflatoxins are stable during food processing, and contaminated food must be discarded causing significant economic losses amounting to billions of US$ in the US alone (Robens and Cardwell, 2003).

Antibodies against different *Aspergillus* antigens have been used to track infections by staining the fungal cell wall (Ste-Marie et al., 1990; Hao et al., 2008; Kumar and Shukla, 2015; Schubert et al., 2018). Other *Aspergillus*-specific antibodies have been used to detect allergens (Kurup and Banerjee, 2000) and disease-related biomarkers (Thornton, 2010) released by pathogenic strains, and to detect aflatoxin contamination in agricultural products (Wacoo et al., 2014). Fungal-type galactomannan (GM) is a heat-stable heteropolysaccharide and a major component of *Aspergillus* cell walls. It comprises a linear mannan core and short, branched β-1,5-linked galactofuranose (Gal*f* ) chains (Latge et al., 1994). Antibodies that recognize GM, the main biomarker of IA, are commercially available (Thornton, 2010). Most *Aspergillus*-specific antibodies are generated using undefined preparations, such as crude extracts, so the precise antigens are often unknown. This makes it difficult to characterize the antibodies in detail and limits their commercial applications. We have generated monoclonal antibodies (mAbs) against several different intracellular and extracellular antigens of aflatoxigenic *A. flavus* and *A. parasiticus* using crude cell wall antigen preparations (Schubert et al., 2018). Here we describe the generation and antigen-specific characterization of mAb AP3, its potential suitability for the rapid serological detection of IA, and possible further commercial applications.

## Materials and Methods

### Fungal Strains

Pure cultures of *A. flavus, A. parasiticus, A. nidulans, A. niger, Fusarium oxysporum, F. culmorum, Phytophthora nicotianae, Rhizoctonia solani, Pythium ultimum, Botrytis cinerea, Cercospora nicotianae, Thielaviopsis basicola*, and *Penicillium chrysogenum* were obtained from the German Collection of Microorganisms and Cell Cultures (DSMZ, Braunschweig, Germany) and maintained on potato dextrose agar (PDA; Carl Roth, Karlsruhe, Germany), tomato agar (25% (v/v) tomato juice, 3 g/l CaCO_3_, 15 g/l agar) or liquid potato dextrose broth medium (PDB; Carl Roth). The wild-type *A. fumigatus* strain D141 (Reichard et al., 1990) and Gal*f*-deficient mutant Δ*glfA* were cultivated as previously described (Schmalhorst et al., 2008). The *F. oxysporum* strain DSM 62316 used for immunofluorescence analysis and enzyme-linked immunosorbent assay (ELISA) experiments was cultivated and prepared as previously reported (Wiedemann et al., 2016).

### Preparation of Fungal Antigens

#### Cell Wall Fragments and Cell Wall Proteins

*Aspergillus* conidia were isolated and used to inoculate liquid cultures in PDB or Czapek Dox medium. For all other fungi, an overgrown agar slice was used to inoculate liquid cultures based on the media and cultivation conditions recommended by the DSMZ. Harvested mycelia were disrupted under liquid nitrogen using a mortar and pestle to obtain cell wall fragments (CWFs) and were washed three times in 1 M NaCl to remove cytosolic antigens (Pitarch et al., 2008).

CWFs representing each fungal species listed above were resuspended in deionized water, lyophilized and weighed. Cell wall-associated proteins (CWPs) were extracted from *A. flavus* (AF-CWPs) and *A. parasiticus* CWFs (AP-CWPs) using a reducing extraction buffer (50 mM Tris-HCl pH 8.0, 0.1 M EDTA, 2% (w/v) SDS, 10 mM DTT) and resuspended in 1× phosphate-buffered saline (PBS; 137 mM NaCl, 2.7 mM KCl, 8.1 mM Na_2_HPO_4_, 1.5 mM KH_2_PO_4_, pH 7.4) as previously described (Prados-Rosales et al., 2009).

#### Preparation of Extracellular Aspergillus Antigens and GM Supernatants

Extracellular *Aspergillus* antigens secreted during growth were prepared by inoculating 400 ml Czapek Dox medium with 10^6^ *A. flavus* conidia/ml and removing the mycelia after 7 days of growth at 28°C by filtering through three layers of Miracloth (Merck, Darmstadt, Germany). The supernatant was then precipitated in 2.5 volumes of ethanol overnight at 4°C, and the pellet was collected by centrifugation (3,000 g, 10 min, 4°C). The precipitate was washed three times with ethanol and resuspended in water, then freeze-dried and stored at −20°C. The protein content was determined using the Roti-Quant Bradford assay (Carl Roth). Supernatants from the *A. fumigatus* Δ*glfA* mutant, and GM-containing supernatants from *A. fumigatus* strain D141 (SD-Asp) and *F. oxysporum* strain DSM 62316, were prepared as previously described (Wiedemann et al., 2016).

### Antibody Generation and Purification

Five 6-weeks-old female BALB/c mice (veterinary license: 9.93.2.10.54.07.044) were intraperitoneally immunized with 150 μg *A. parasiticus* CWFs in a total volume of 100 μl prepared with Gerbu Adjuvant MM (Gerbu Biotechnik, Heidelberg, Germany), and subsequent boosts were carried out at 2-weeks intervals. Five days after the final boost (boost six), B-lymphocytes were isolated from spleens and fused to myeloma cells (SP2/mIL6). The resulting hybridoma cells were cultivated in Gibco RPMI GlutaMAX medium (Thermo Fisher Scientific, Waltham, MA, USA) and the supernatants of cells producing *Aspergillus*-specific antibodies were screened by ELISA using a goat anti-mouse Fc antibody for the selection of IgG antibodies. Positive hybridoma cells were singularized by limiting dilution, and monitored using the Cellavista imaging system (Roche, Basel, Switzerland). Stable cell line AP3 (producing mAb AP3) was maintained for long-time storage by cryopreservation in liquid nitrogen. The isotype of this cell line was determined using a mouse immunoglobulin isotyping kit (BD Biosciences, San Jose, CA, USA). The cells were transferred to serum-free H5000 medium (PAN-Biotech, Aidenbach, Germany) and incubated continuously for up to 2 months at 37°C in a 5% CO_2_ atmosphere in a CELLine bioreactor flask CL1000 (Sigma-Aldrich, St. Louis, MO, USA).

The AP3 antibody was purified by passing the hybridoma supernatant through MEP HyperCel resin (Pall, Port Washington, NY, USA) using the ÄKTAexplorer 10 fast protein liquid chromatography (FPLC) system (GE Healthcare, Munich, Germany). The purified antibody was dialyzed against PBS, supplemented with 0.02% (w/v) NaN_3_ and stored at 4°C. Biotinylated mAb AP3 was prepared using the EZ-Link biotinylation kit (Thermo Fisher Scientific) according to the manufacturer's instructions.

### Recombinant Protein Production

The cDNA coding for the *A. flavus* mycelial catalase (XP_002380889.1) was synthesized with codon optimization for *Escherichia coli* and transferred to the bacterial expression vector pET-22b(+) (Novagen, Darmstadt, Germany), providing sequences for an N-terminal signal peptide (targeting the periplasmic space) and a C-terminal His_6_ tag (for affinity purification and detection). The construct was introduced into competent *E. coli* BL21(DE3) cells (New England Biolabs, Frankfurt am Main, Germany) and a positive clone of *A. flavus* mycelial catalase was selected for production and purification by Ni-NTA affinity chromatography, according to the manufacturer's instructions (Novagen).

### One-Dimensional Electrophoresis (1DE) and Immunoblot

AF-CWPs and precipitated extracellular *Aspergillus* antigens were boiled in 5× reducing SDS loading buffer (62.5 mM Tris-HCl pH 6.8, 10% (v/v) 2-mercaptoethanol, 4% (w/v) SDS, 30% (w/v) glycerol, 0.05% (w/v) bromophenol blue), and separated by discontinuous SDS-PAGE using a 12% (w/v) polyacrylamide separating gel. Proteins were visualized using Coomassie Brilliant Blue (Fairbanks et al., 1971). The separated proteins were transferred to 0.45-μm nitrocellulose membranes by electroblotting, and free binding sites were blocked with 3% (w/v) milk powder in PBS containing 0.05% (v/v) Tween-20 (PBS-T). After each step, the membrane was washed with PBS-T. Proteins were detected with the purified mAb AP3 (2 μg/ml) and an alkaline phosphatase (AP)-labeled goat anti-mouse Fc (GAM^AP^ Fc, 160 ng/ml) (Jackson Immunoresearch Laboratories, West Grove, PA, USA). After washing in PBS-T and equilibrating in AP buffer (100 mM Tris-HCl pH 9.6, 100 mM NaCl, 5 mM MgCl_2_), the signal was detected by incubating in AP buffer containing nitro-blue tetrazolium and 5-bromo-4-chloro-3-indolylphosphate (NBT/BCIP) diluted 1:100.

### Two-Dimensional Electrophoresis (2DE), Immunoblot and Mass Spectrometry

*Aspergillus* spores were germinated in Czapek Dox medium (28°C, 16 h). Young *A. flavus* mycelia were ground to a fine powder under liquid nitrogen, and the proteins were precipitated by adding 1.8 ml ice-cold acetone containing 0.7% (v/v) 2-mercaptoethanol. The samples were incubated at −20°C for at least 1 h and then centrifuged (13,000 g, 20 min, 4°C). The pellets were resuspended in ice-cold acetone plus 0.7% (v/v) 2-mercaptoethanol followed by incubation and centrifugation as above. After the second centrifugation step, the pellets were washed twice in ice-cold acetone without 2-mercaptoethanol, dried at room temperature, and stored at −20°C. The proteins in the pellets were resolubilized overnight at room temperature in isoelectric focusing buffer (7 M urea, 2 M thiourea, 2% (w/v) CHAPS, 30 mM Tris-HCl pH 8.8), and the mixture was centrifuged as above to remove debris. The protein content of the supernatant was quantified using the 2D quant kit (GE Healthcare), and two 100-μg CWP aliquots were labeled with 200 pmol Cy3 (GE Healthcare) according to the manufacturer's instructions.

Gel electrophoresis was carried out as previously described (Horn et al., 2013). Two 2D gels were prepared under the same conditions—the first one was used for immunoblot analysis as described above, the second one as a preparative gel for the identification of proteins by mass spectrometry. After protein separation, both gels were scanned using the Ettan DIGE Imager (GE Healthcare) with the filter for Cy3 to localize all protein spots and to enable matching. Fungal proteins recognized by mAb AP3 were detected by first probing with AP3 (400 ng/ml) followed by a goat anti-mouse Cy5-labeled antibody (120 ng/ml). The membrane was then scanned twice using the Cy3 and Cy5 filters to reveal the positions of proteins bound by the AP3 antibody. The images were processed with DeCyder v7.0 (GE Healthcare). Spots of interest were marked on the preparative gel followed by blind picking. The proteins in gel spots were alkylated and digested with trypsin (Promega, Mannheim, Germany) before identification by mass spectrometry as previously described (Spiegel et al., 2015). The raw data files were evaluated using the NCBI *A. flavus* reference database (12,587 sequences; 5,779,766 residues).

### Immunofluorescence Microscopy

Round glass coverslips were washed in 70% (v/v) ethanol, coated with 0.1% (v/v) poly-L-lysine and air dried. The coverslips were then washed in deionized water and deposited in 12-well cell culture plates, which were blocked with 3% (w/v) milk powder in PBS-T. Germinated *A. flavus* and *A. parasiticus* conidia (overnight incubation in RPMI medium at 37°C) were added to the wells, and the plates were centrifuged (2,000 g, 15 min, room temperature) to deposit the germlings onto the coverslips. For direct staining, mAb AP3 (2 μg/ml) was added to the wells and incubated for 2 h at room temperature. After washing with PBS-T, AP3 binding to conidia and short hyphae was detected by adding 1.5 μg/ml GAM^Dylight^ 594 H+L (Jackson ImmunoResearch Laboratories) and incubating for 1 h at room temperature. The round coverslips were then placed upside down on a slide and sealed with nail polish to prevent desiccation. Samples were analyzed with a Leica DMR fluorescence microscope (Leica Microsystems, Wetzlar, Germany) using excitation/emission maxima of 592/617 nm. *A. fumigatus* D141, Δ*glfA* and *P. chrysogenum* were detected with mAb AP3 and suitable Cy3-labeled secondary antibodies (Jackson ImmunoResearch Laboratories). Images were captured using a Leica SP-5 confocal laser scanning microscope (Leica Microsystems) as previously described (Wiedemann et al., 2016).

### Periodate Oxidation

Extracellular *Aspergillus* antigens and CWPs from *A. flavus* were immobilized on ELISA plates (40 μg/well) and treated with 200 μl sodium *meta*-periodate buffer (20 mM NaIO_4_ in 50 mM sodium acetate buffer, pH 4.5) for 16 h in darkness at 4°C. The periodate oxidation of the SD-Asp antigen was carried out as previously described (Thornton, 2008). The binding of mAb AP3 (400 ng/ml) to the antigen after periodate treatment was measured by ELISA as described below. Untreated *Aspergillus* antigens were used as a positive control and PBS as a negative control.

### Digestion of *Aspergillus* Antigens

Extracellular *Aspergillus* antigens and *A. flavus* CWPs (40 μg) were dissolved in 50 mM ammonium bicarbonate buffer supplemented with 1.5 μg trypsin protease (Promega). The solution was then incubated at 37°C overnight and the antigen (20 μg) was separated by SDS-PAGE using a 12% (w/v) polyacrylamide separating gel. The binding of AP3 to the protease-treated antigens was analyzed by immunoblot as described above.

### ELISA

#### Quantification of mAb AP3

The concentration of AP3 was determined by sandwich ELISA using an IgG1 standard (Rasche et al., 2011) to generate a calibration curve. A high-binding microtiter plate (Greiner Bio-One, Frickenhausen, Germany) was coated with 300 ng/ml goat-Fab anti-mouse Fab in PBS (Jackson ImmunoResearch Laboratories) overnight at 4°C. After each step, the plates were washed with 200 μl PBS-T. After blocking with 200 μl 3% (w/v) skimmed milk in PBS-T for 1 h, several dilutions of AP3 (0.4–100 ng/ml) were loaded onto the ELISA plate adjacent to the calibration curve of the standard IgG1. Binding was detected by adding 160 ng/ml horseradish peroxidase (HRP)-labeled goat anti-mouse Fc antibody (Jackson ImmunoResearch Laboratories) and 2,2′-azino-*bis*(3-ethylbenzthiazoline-6-sulfonic acid) (ABTS) as the substrate. After 30 min incubation at room temperature, the absorbance was determined by spectrophotometry at 405 nm. After each incubation step, the microtiter plate was washed three times with PBS-T. All measurements were taken in triplicate.

#### Reactivity of mAb AP3 to Fungal Antigens

The specificity of mAb AP3 binding was measured by direct ELISA with CWFs. The prepared CWFs were used to coat high-binding microtiter plates (150 μg/ml in water) overnight at 37°C. After blocking as described above, several concentrations of AP3 (0.002–2 μg/ml) were applied as the primary antibody, followed by detection as described above. The effect of periodate oxidation was evaluated by coating high-binding microtiter plates with periodate-treated *A. flavus* CWPs (2 μg) and GM standard SD-Asp (1:100), and using untreated *Aspergillus* antigens as a control. After blocking as described above, purified AP3 (400 ng/ml) was applied as the primary antibody, followed by detection as described above.

Double antibody sandwich ELISA (DAS-ELISA) was carried out to detect CWPs and extracellular antigens secreted by *A. flavus*. High-binding microtiter plates were coated with mAb AP3 (400 ng/ml) overnight at 4°C. After blocking with 2% (w/v) biotin-free bovine serum albumin (BSA; Carl Roth), 100-μl aliquots of serially-diluted *A. flavus* antigens (0.005–25 μg/ml) were applied for 1 h and detected using biotinylated mAb AP3 (600 ng/ml) and AP-labeled streptavidin (200 ng/ml) (Jackson ImmunoResearch Laboratories), followed by detection as described above.

To analyze *Aspergillus* supernatants by direct-coating ELISA, the culture supernatants from *A. fumigatus* wild-type D141 (SD-Asp, 1:10–1:100) and Δ*glfA* (1:10) were coated onto high-binding microtiter plates for 1 h at room temperature. After blocking as described above, purified AP3 (400 ng/ml) or the GM-specific antibody IgM L10-1 (Heesemann et al., 2011) and the IgM AB135-8 (Wiedemann et al., 2016), which recognizes a novel Gal*f*-containing antigen, were applied as primary antibodies, followed by detection using a secondary HRP-labeled goat anti-mouse IgG or goat anti-mouse IgM. The signal was developed by incubating in ABTS for 20 min and detected by measuring the absorbance at 405 nm. Measurements were taken in triplicate.

Sandwich ELISA was used to detect the presence of GM in serum. Purified AP3 (400 ng/ml), L10-1, or AB135-8, were coated onto a high-binding microtiter plate at 4°C overnight. After blocking as described above, 100 μl GM-positive serum from the Platelia *Aspergillus* enzymatic immunoassay (EIA) kit (Bio-Rad, Hercules, CA, USA) was diluted 1:5,000 or 1:100,000, applied for 1 h and developed using the Gal*f*-specific EB-A2 conjugate and the detection reagents provided in the kit. The reaction was stopped after 30 min and the absorbance was measured at 450 nm. Measurements were taken in triplicate.

To investigate the cooperation of L10-1 and AP3 as capture and detection antibodies in a sandwich ELISA, purified AP3 or L10-1 (each 900 ng/ml) were coated onto a high-binding microtiter plate at 4°C overnight. After blocking as described above, serially-diluted CWPs or extracellular antigens (1:320–1:20,480) from *A. flavus* were applied for 1 h, and bound antigens were detected using the corresponding detection antibody: AP3 or L10-1 (each 900 ng/ml). Binding was detected using a secondary HRP-labeled goat anti-mouse IgG or goat anti-mouse IgM. Absorbance was measured at 405 nm after 40 min incubation in ABTS substrate. Measurements were taken in triplicate.

#### Glycoarray

The carbohydrate specificity of mAb AP3 was determined using a thematic glycoarray as previously described (Krylov et al., 2018b; Matveev et al., 2018). To 96-well streptavidin-coated plates (Pierce, Waltham, MA, USA), we added 20 pmol/well of the biotin-tagged oligosaccharides 1–13 (Argunov et al., 2015, 2016; Krylov et al., 2018a) in 100 μl PBS containing 0.05% (v/v) Tween-20 and 0.1% (w/v) BSA. The plates were then incubated for 2 h at 37°C before adding mAb AP3 serially diluted in the same buffer (1000, 250, 50 and 10 ng/ml) and incubating for another 1 h at 37°C. After washing, we added a rabbit anti-mouse IgG HRP conjugate (Imtek, Moscow, Russia) and incubated for 1 h at 37°C. After washing three times, color development was initiated by adding 100 μl TMB monocomponent substrate for 15 min and stopped by adding 50 μl 1 M sulfuric acid. Absorbance was measured at 450 nm using a MultiSkan GO plate reader (Thermo Fisher Scientific). Measurements were carried out twice in triplicate, and results are presented as means ± SD.

### Statistical Analysis

Significant differences between the antigen-binding and control treatments in ELISAs and glycoarray experiments were determined by one way analysis of variance (ANOVA) followed by *post-hoc* Bonferroni testing using Excel software (Microsoft, Redmond, Washington, USA). Significant differences in antigen binding between ELISA experiments were confirmed by Student's *t*-test, with the significance threshold set at *p* < 0.05.

## Results

### Characterization of the *Aspergillus*-Specific mAb AP3

Following the immunization of mice with *A. parasiticus* cell wall fragments (CWFs), seven hybridoma clones producing *Aspergillus*-specific IgG antibodies were selected by ELISA. The hybridoma clone that showed the strongest reactivity against *A. parasiticus* CWFs was selected for further analysis, and the corresponding mAb (AP3) was assigned to isotype IgG1 and κ. Because CWFs and extracted cell wall proteins (CWPs) comprise a mixture of antigens of different sizes and chemical compositions, the precise antigen bound by AP3 was initially unknown.

**Figure 1:**
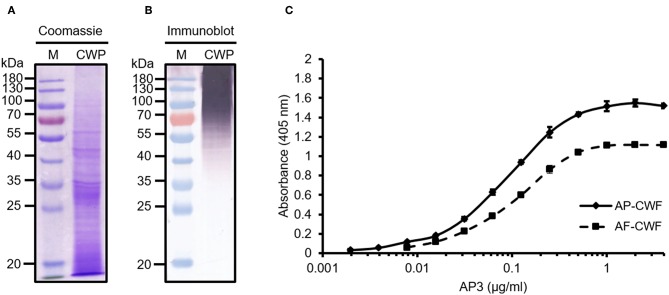
Analysis of *A. flavus* cell wall proteins (CWPs) and the specific detection of cell wall fragments (CWFs) by ELISA using mAb AP3. Extracted CWPs were separated by SDS-PAGE and stained with **(A)** Coomassie Brilliant Blue or **(B)** transferred to a nitrocellulose membrane. Immunoblot detection was carried out using 200 μl culture supernatant from monoclonal hybridoma cell line AP3 and GAM^AP^ Fc (120 ng/ml) followed by visualization using NBT/BCIP. M: Pre-stained protein marker (Fermentas). **(C)** For the ELISA, 150 μg/ml CWFs from *A. flavus* (AF-CWF) and *A. parasiticus* (AP-CWF) were coated onto a microtiter plate. After blocking free binding sites with 3% (w/v) skimmed milk, bound antigens were detected using purified mAb AP3 (0.008–4 μg/ml) and an HRP-labeled goat anti-mouse Fc antibody (160 ng/ml). Absorbance was measured in triplicate after 15 min substrate incubation.

First we analyzed the ability of AP3 to bind to *A. flavus* CWPs by immunoblot. The CWPs were separated by SDS-PAGE (Figure 1A) and the proteins were transferred to a membrane and probed with AP3. The antibody bound to multiple undefined bands with molecular masses exceeding 35 kDa and was particularly reactive against *A. flavus* proteins with molecular masses exceeding 70 kDa (Figures 1B, 3A). This provided the first evidence that the epitope recognized by AP3 is shared by multiple glycoproteins.

The reactivity of AP3 against *Aspergillus* antigen preparations and its cross-reactivity with other fungal plant pathogens was also tested by ELISA. The antibody bound to CWFs prepared from *A. flavus, A. parasiticus, A. nidulans* and *A. niger* (Figure 1C; Table 1), as well as CWPs extracted from *A. flavus* and *A. parasiticus*, but not to other fungal preparations (Table 1). This probably reflects the ability of AP3 to detect an epitope that is conserved in the genus *Aspergillus* but absent in the other fungal genera tested in this experiment (Table 1).

**Table 1:** Cross-reactivity of mAb AP3 against CWFs from different fungal pathogens measured by ELISA.

**Species**	**Source**	**Binding of mAb AP3**
*Aspergillus flavus* Link:Fries	DSMZ 818	+++
*Aspergillus parasiticus* Speare	DSMZ 1300	+++
*Aspergillus nidulans* (Eidam) Winter	DSMZ 820	++
*Aspergillus niger* van Tieghem	IME	+
*Fusarium oxysporum* f. sp. *nicotianae*	IME	–
*Fusarium culmorum* W. G. Smith	IME	–
*Phytophthora nicotianae*	DSMZ 1828	–
*Rhizoctonia solani* Kühn	IME	–
*Pythium ultimum* Trow	DSMZ 62987	–
*Botrytis cinerea* Persoon:Fries	IME	–
*Cercospora nicotianae*	IME	–
*Thielaviopsis basicola*	IME	–

*CWFs (150 μg/ml in water) were coated overnight at 37°C. After blocking free binding sites with 3% (w/v) skimmed milk, the bound antigen was detected using purified mAb AP3 (400 ng/ml). Antibody binding to fungal CWFs was confirmed by the addition of GAM^HRP^ Fc (1:5,000). Absorption at 405 nm was measured after 30 min incubation with the substrate ABTS. Cross-reactivity was determined based on the extinction levels after subtracting the background signal. DSMZ, German Collection of Microorganisms and Cell Cultures, Leibniz Institute, Braunschweig, Germany; IME, Fraunhofer IME, Aachen, Germany; –, OD_405 nm_ < 0.1; +, OD_405 nm_ = 0.50–0.99; ++, OD_405 nm_ = 1.00–1.49; + + +, OD_405nm_ > 1.5*.

**Figure 2:**
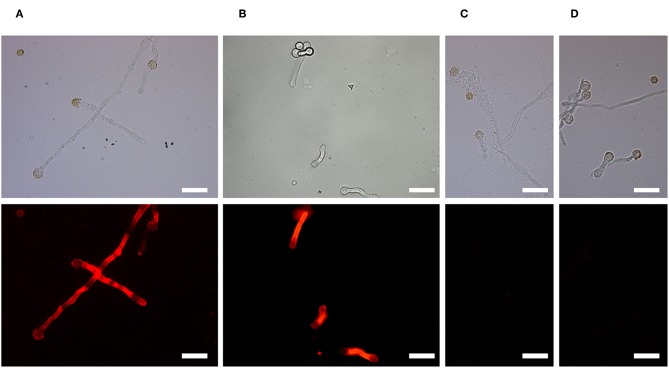
Binding of mAb AP3 to *A. parasiticus* and *A. flavus* revealed by immunofluorescence microscopy. **(A)** *A. parasiticus*. **(B)** *A. flavus*. **(C)** *A. parasiticus* control. **(D)** *A. flavus* control. Mycelia were immobilized on glass coverslips and incubated with 200 μl hybridoma supernatant containing 25 μg/ml mAb AP3 or (Rasche et al., 2011; unrelated control). The binding of AP3 to its antigen was verified using GAM^Dylight^ 594 H + L (1.5 μg/ml). Antibody–antigen complexes were visualized under a Leica DMR fluorescence microscope. For comparison, light microscope images are shown above the immunofluorescence images. Scale bar = 50 μm.

Immunofluorescence microscopy showed that the antigen recognized by AP3 is located on the *Aspergillus* cell surface (Figure 2), particularly the hyphal walls and tips of the germination tubes in *A. parasiticus* (Figure 2A), *A. flavus* (Figure 2B), and *A. fumigatus* (Supplemental Figures 1A,B) compared to control samples (Figures 2C,D; Supplemental Figures 1E,F).

**Figure 3:**
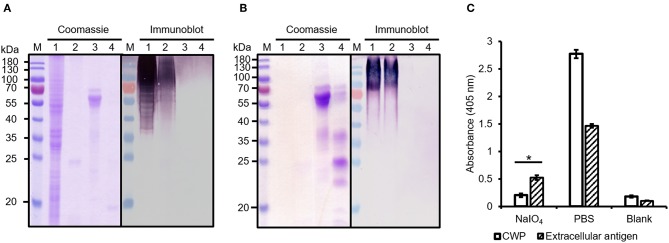
Analysis of protease-treated and periodate-treated *Aspergillus* antigens. **(A)** Cell wall proteins and **(B)** extracellular secreted antigens of *A. flavus* were extracted and digested with trypsin. Fractions were separated by SDS-PAGE and stained with Coomassie Brilliant Blue. Proteins transferred to a nitrocellulose membrane were detected with a purified mAb AP3 (400 ng/ml) and an AP-labeled goat anti-mouse antibody (120 ng/ml) followed by NBT/BCIP substrate incubation. M: Protein marker (Fermentas); Lane 1: sample without trypsin treatment; 2: sample with trypsin; 3: control protein fetuin without trypsin treatment; 4: control protein fetuin with trypsin. **(C)** Prepared *A. flavus* cell wall proteins (CWPs) and extracellular antigens (40 μg/well) were coated onto a microtiter plate and oxidized with periodate (80 mM). Untreated preparations were used as controls. After blocking free binding sites with 3% (w/v) skimmed milk, bound antigens were detected with mAb AP3 (400 ng/ml) and an HRP-labeled goat anti-mouse Fc antibody (120 ng/ml). Absorbance was measured in triplicate after 20 min substrate incubation. Values represents means ± SD (*n* = 3) for treated (NaIO_4_), untreated (PBS), and control (Blank). An asterisk denotes a statistically significant reduction in antigen binding between treated and untreated samples (CWF and extracellular antigen, *p* < 0.05).

We next investigated the ability of mAb AP3 to bind extracellular antigens precipitated from the *A. flavus* culture supernatant (Figure 3B) and *A. fumigatus* spent culture medium (SD-Asp) (Supplemental Figure 2A) by immunoblot and ELISA. Compared to the protein-rich cell wall fraction (Figure 3A), the extracellular fraction contained mainly GM and small amounts of protein, hence no distinct protein band was observed in the Coomassie-stained gel (Figure 3B). The low protein content of the extracellular fraction was also confirmed by Bradford assay (data not shown). However, more sensitive immunoblot analysis revealed multiple undefined bands with molecular masses exceeding 70 kDa (Figure 3B), indicating that glycoantigens sharing the epitope recognized by AP3 are also present in the culture supernatant. The strong binding observed by direct-coating ELISA (Figure 3C; Supplemental Figure 2A) confirmed that the antigens detected by AP3 are present in both the culture supernatant and the *Aspergillus* cell wall.

### Identification of the AP3 Antigen

**Figure 4:**
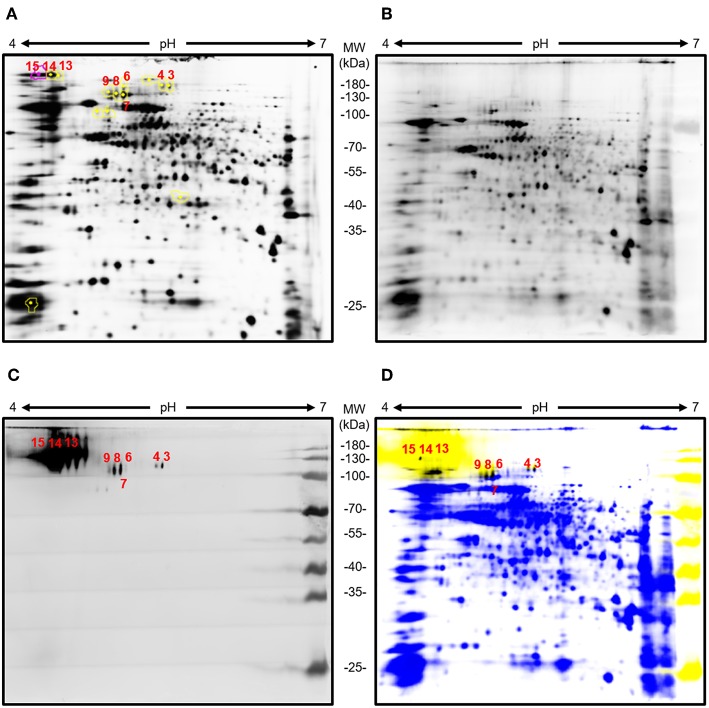
Analysis of young *A. flavus* mycelia cell wall proteins by 2DE and immunoblot with AP3 as the detection antibody. Total *Aspergillus* mycelia and CWPs (100 μg) were labeled with Cy3 and separated in the first dimension on a 7-cm pH 4–7 immobilized pH gradient strip followed by separation in the second dimension by 12.5% (v/v) SDS-PAGE. Separated *Aspergillus* CWPs were transferred onto a nitrocellulose membrane. Immunoblot detection was carried out using purified mAb AP3 (400 ng/ml) and a goat anti-mouse Cy5-labeled antibody (120 ng/ml). Gels and membranes were scanned with an Ettan DIGE Imager equipped with appropriate filters for Cy3 and Cy5, and images were processed with DeCyder software v7.0. **(A)** Cy3 labeled 2D gel of young *A. flavus* proteins. **(B)** Cy3-labeled total CWPs blotted onto a membrane. **(C)** CWPs specifically detected by mAb AP3 visualized using a Cy5-labeled antibody. **(D)** Overlay of Cy3-labeled total CWPs (blue) and AP3-recognized CWPs (yellow). Shared protein spots are highlighted in black. Numbers indicate protein spots selected for analysis by mass spectrometry.

The *Aspergillus* glycoproteins recognized by AP3 were identified by 2DE and mass spectrometry. The separation of young *A. flavus* CWPs by 2DE (Figure 4) revealed a wide range of protein spots with different molecular masses and pI values. However, only a small number of these proteins were detected by immunoblot with mAb AP3 as the probe (Figures 4C,D).

MS/MS analysis revealed five unique proteins in nine spots, indicating that some of the identified proteins were present in multiple spots, possibly due to different forms of post-translational modification (Table 2). Matching the protein sequences against the *A. flavus* NRRL3357 database identified *A. flavus* mycelial catalase, Hsp70, Hsp90, an amidase family protein, and a cell wall glucanase (Table 2). However, these proteins do not share any peptides or sequence similarities. *In silico* analysis revealed that the detected proteins have numerous acceptor sites for *N*-linked and/or *O*-linked glycosylation (Table 2). We therefore tested whether protein glycosylation might be necessary for antigen recognition by mAb AP3. The gene encoding the *A. flavus* mycelial catalase (XP_002380889.1) was produced in *E. coli* BL21 (DE3) cells. Immunoblot analysis revealed that mAb AP3 failed to detect the bacterial recombinant protein, whereas a histidine-specific antibody detected a distinct band with the expected molecular mass of *A. flavus* mycelial catalase (110 kDa) (Supplemental Figure 3). Because *E. coli* does not synthesize eukaryotic-type *N*-linked and *O*-linked glycans, the inability of AP3 to recognize the non-glycosylated mycelial catalase suggests that it binds a carbohydrate epitope present on certain *Aspergillus* glycoproteins or associated with the *Aspergillus* glycosylation pattern, as indicated by the immunoblot data (Figures 1B, 3).

**Table 2:** List of *Aspergillus* proteins detected by mAb AP3.

**Spot no**.	**Short name**	**Protein**	**NCBI reference sequence**	**MW (kDa)**	**pI^a^**	**Score^b^**	**Peptides^c^**	**Sequence coverage (%)**	**Signal peptide^d^**	***N*-glycan acceptor sites^e^**	***O*-glycan acceptor sites^f^**	**Cellular localization**	**Function^g^**
3	Catalase	Mycelial catalase cat1	XP_002380889.1	79.8	5.34	398	18 (13)	21	Yes	4	24	Extracellular (cell wall)	Cell protection (Paris et al., 2003)
4						373	14 (9)	17					
6	Hsp70	Hsp70 chaperone Hsp88	XP_002381416.1	79.8	5.02	680	32 (23)	34	No	3	3	Intracellular	Protein folding (Teutschbein et al., 2010)
8						331	16 (10)	19					
7	Hsp90	Molecular chaperone and allergen Mod-E/Hsp90/Hsp1	XP_002382894.1	79.6	4.97	405	13 (10)	20	No	4	8	Intracellular (cytosolic, cell wall)	Protein folding (Lamoth et al., 2012, 2014, 2016)
9	Amidase	Amidase family protein	XP_002377652.1	60.7	5.22	276	11 (7)	22	Yes	7	9	Extracellular	Unknown
13	Glucanase	Cell wall glucanase (Scw11), putative	XP_002372749.1	61.8	4.63	155	6 (4)	10	Yes	0	122	Extracellular (cell wall)	Cell wall remodeling (Mouyna et al., 2013)
14						262	10 (6)	15					
15						187	8 (5)	11					

*Protein spots were identified by Tandem mass spectrometry and protein sequences were analyzed in silico using the A. flavus NRRL3357 reference database*.

a *Predicted Mw and pI by ExPASy Compute pI/Mw tool*.

b *Protein score in Mascot Search*.

c *Number of total identified peptides/unique peptides*.

d *Predicted signal peptide (ProP 1.0)*.

e *Predicted N-glycosylation sites (NetNGlyc1.0)*.

f *Predicted O-glycosylation sites (NetOGlyc4.0)*.

g *Reference*.

To determine whether AP3 recognizes a carbohydrate or a protein epitope, the CWPs and extracellular secreted antigens were treated with protease or periodate, to remove the protein component and to oxidize the glycans, respectively (Figure 3). No differences in immunoblot profiles were observed when we compared untreated controls with *A. flavus* CWPs (Figure 3A) and extracellular antigens (Figure 3B) digested with trypsin. This might reflect the location of trypsin cleavage sites toward the N-terminus of the target protein, resulting in minor mass changes, or proteolytic stability caused by the presence of glycans around the peptide backbone of the amino acids adjacent to the glycosylation site, thus preventing the contact between the glycoprotein surface and the protease active site (Sola and Gribenow, 2009). The epitope recognized by AP3 was found to be periodate sensitive (*p* < 0.05) and therefore most likely a carbohydrate (Figure 3C). Our results therefore demonstrate that AP3 recognizes an *Aspergillus* glycoantigen rather than a peptide or protein epitope. Furthermore, the smear-like staining observed for the extracellular fraction in the immunoblot suggests that most of the AP3 antigens are fragments of cell wall carbohydrate polymers.

**Figure 5:**
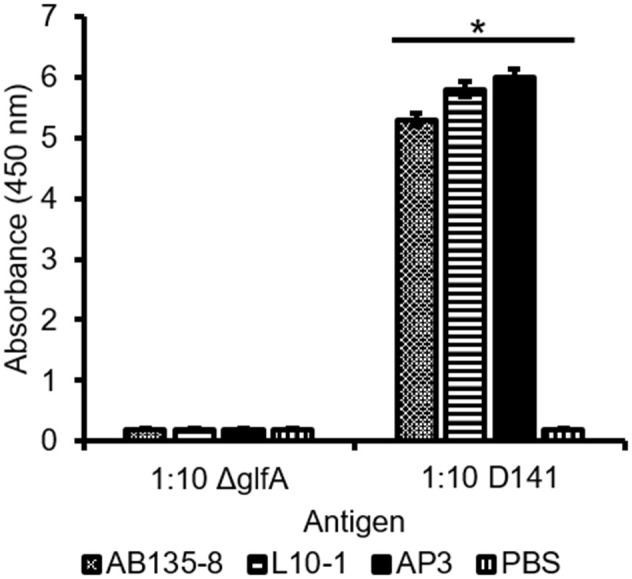
Detection of *A. fumigatus* D141 GM and Δ*glfA* supernatant by ELISA. Culture supernatants from *A. fumigatus* Δ*glfA* and wild-type D141 strains were coated (1:10) onto a microtiter plate. GM was detected using 400 ng/ml purified mAb AP3 or 400 ng/ml of the IgMs L10-1 and AB135-8, which recognize distinct Gal*f*-dependent antigens, followed by isotype-specific antibodies labeled with HRP. Data are means ± SD (*n* = 3). An asterisk denotes a statistically significant difference in antigen binding between the D141 and Δ*glfA* strain (*p* < 0.005).

Taken together, our data suggested that AP3 recognizes a major carbohydrate of the *Aspergillus* cell wall, which is homogenously distributed on the hyphal surface, found in a speckled pattern on swollen conidia, and not present on the surface of resting conidia. This pattern resembles that of GM (Heesemann et al., 2011) and we therefore tested the Gal*f*-deficient *A. fumigatus* mutant Δ*glfA* (Schmalhorst et al., 2008). Immunofluorescence microscopy revealed that the hyphae of the parental *A. fumigatus* strain D141 were completely stained (Supplemental Figure 4A), but AP3 did not bind to the Δ*glfA* mutant (Supplemental Figures 1C,D), indicating that Gal*f* residues are part of the epitope. Moreover, the double-staining of *A. fumigatus* hyphae with AP3 (IgG) and the GM-specific antibody L10-1 (IgM) demonstrated that the staining patterns of both antibodies are similar (Supplemental Figure 4). This prompted us to analyze *Penicillium chrysogenum*, a non-pathogenic mold known to produce GM. AP3 decorated the hyphal surface of *P. chrysogenum* and additional material bound to the glass surface in close proximity to the hyphae (Supplemental Figures 5A,B). This staining pattern suggests that AP3 recognizes structurally identical GM antigens that are present on the surface of this fungus and partially released into the surrounding medium. We performed additional tests on culture supernatants derived from the *A. fumigatus* wild-type strain D141 and Δ*glfA* mutant for further characterization of AP3 by ELISA (Figure 5). Supernatants diluted 1:10 in PBS were coated onto the ELISA plate and probed with AP3, the GM-specific IgM L10-1, and AB135-8, an IgM that recognizes a Gal*f* antigen found prominently in the *Fusarium* cell wall but present in only limited amounts in the *Aspergillus* cell wall (Wiedemann et al., 2016). All three antibodies recognized their antigen in the culture supernatant of the wild-type strain, but not in the supernatant of the Δ*glfA* mutant (Figure 5). Similar results were observed for AP3 and L10-1 at a dilution of 1:100 (Supplemental Figure 2A).

### Antibody-Based GM Detection and Specificity of mAb AP3

We next investigated whether AP3 and L10-1 (Heesemann et al., 2011) can cooperate to bind the soluble fungal-type GM of *A. fumigatus* (SD-Asp) (Supplemental Figure 2B), *A. flavus* extracellular antigens, and *A. flavus* cell wall proteins (AF-CWPs) (Supplemental Figures 2C,D). We therefore carried out a sandwich ELISA in which AP3 was the capture reagent and L10-1 the detection reagent or vice versa, and the detection reagent was in turn bound by an HRP-labeled anti-mouse isotype-specific secondary antibody. As shown in Supplemental Figures 2B,D, L10 detected all tested Gal*f*-containing antigens that were captured by AP3. Similarly, AP3 recognized CWPs immobilized on the solid phase by L10-1. However, the extracellular antigens of *A. flavus* were not captured by L10-1 antigens (Figure 2C). Given the diverse structures of Gal*f*-antigens in *Aspergillus* species, these results provide further evidence that AP3 recognizes a defined Gal*f* epitope that may differ slightly from that recognized by L10-1. Moreover, these findings suggest that mAb AP3 detects a Gal*f* epitope that is present less frequently in extracellular *Aspergillus* antigens compared to CWPs from *A. flavus*.

**Figure 6:**
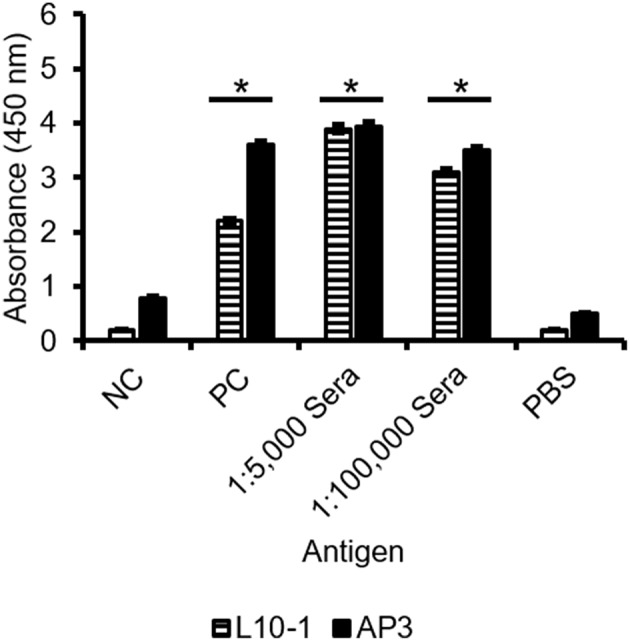
Detection of GM in sera by sandwich ELISA using GM-specific antibodies. Microtiter plates were coated with 400 ng/ml purified mAb AP3 and L10-1. After blocking free binding sites with 3% (w/v) skimmed milk, sera diluted 1:5,000 or 1:100,000 were applied and detected using the EB-A2 conjugate. The signal was measured in triplicate at 450 nm after 20 min substrate incubation (ABTS). PC: *Aspergillus*-positive control serum from the Platelia *Aspergillus* EIA. NC: negative control serum from the Platelia *Aspergillus* EIA. PBS: negative control. An asterisk denotes a statistically significant difference in antigen binding relative to the NC control serum (*p* < 0.005).

GM is the most important immunological biomarker of invasive aspergillosis (IA). Therefore, the ability of AP3 to detect *Aspergillus* GM in serum was compared to the Gal*f*-specific IgM L10-1 (Figure 6). In a sandwich ELISA format, AP3, and L10-1 were coated onto the ELISA plate. After incubation with the positive control serum, the wells were incubated with HRP-labeled EB-A2 (a Gal*f*-specific IgM antibody) according to the procedure of the Platelia *Aspergillus* EIA. As shown in Figure 6, AP3 generated significantly (*p* < 0.05) stronger signals than L10-1, which demonstrates the potential of AP3 as a candidate diagnostic antibody for IA.

**Figure 7:**
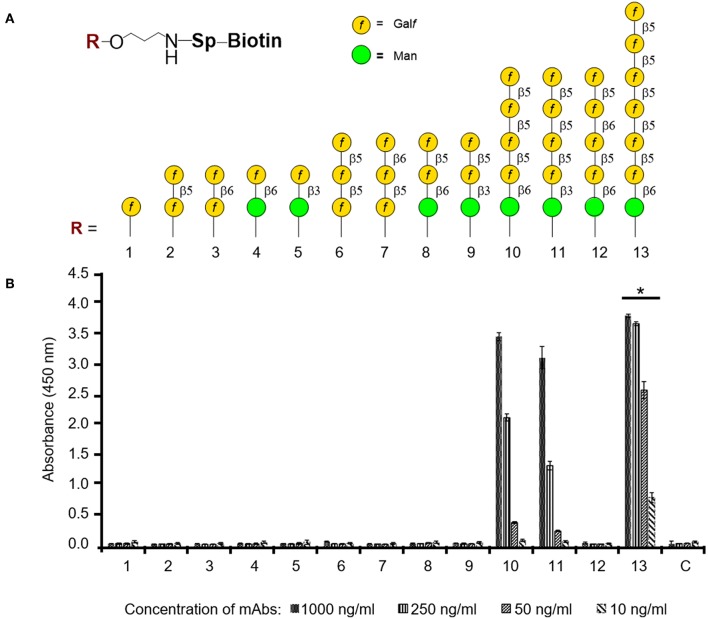
ELISA to determine the oligosaccharide specificity of mAb AP3. **(A)** Composition of thematic glycoarray oligosaccharide ligands representing key structural elements of GM. The carbohydrate sequences are represented as previously reported (Varki et al., 2009). **(B)** Carbohydrate specificity of antibody AP3. Biotin-tagged oligosaccharides 1–13 were applied to a streptavidin-coated microtiter plate and detected with mAb AP3 (1,000, 250, 50, and 10 ng/ml) and anti-mouse IgG HRP conjugate followed by TMB substrate incubation. All measurements were taken twice in triplicate. Asterisk denotes the statistically significant difference in binding between AP3 and heptamer 13 compared to pentamers 10 and 11 (*p* < 0.05).

The epitope specificity of mAb AP3 was investigated using a library of 13 synthetic oligosaccharides representing distinct fragments of *Aspergillus* GM (Argunov et al., 2015, 2016; Krylov et al., 2018a), differing in length and in the nature of the linkages between monosaccharide residues (Figure 7A). AP3 showed the highest affinity (*p* < 0.05) for heptamer **13**, which contains a hexameric block of β-1,5-linked Gal*f* residues (Figure 7B), and lower affinity for pentamers **10** and **11**, containing four β-1,5-Gal*f* units linked to a terminal Man*p* residue via β-1,6 or β-1,3 bonds. There was no affinity for pentamer **12** with β-1,6 linkages between Gal*f* residues, representing structures recently discovered in *A. fumigatus* GM (Kudoh et al., 2015; Krylov et al., 2018a), nor for trimer **6**, comprising three β-1,5-Gal*f* units. Taken together, these data indicate show that AP3 binds specifically to a tetramer of β-1,5-Gal*f* units present in *Aspergillus* GM and Gal*f*-containing *Aspergillus* glycoproteins. The binding of AP3 to *P. chrysogenum* indicates the presence of an identical structure in this species (Supplemental Figure 5).

## Discussion

### Identification of the Epitope Recognized by mAb AP3

The fungal cell wall is a complex and dynamic structure that provides protection and mediates interactions with the environment. Detailed investigations of the composition and biosynthesis of the *A. fumigatus* cell wall have identified carbohydrates, such as chitin, glucans, and GM as major structural components. The cell wall is adorned with CWPs that can be attached to the plasma membrane by glycosylphosphatidylinositol (GPI) anchors or linked to glucan structures, thus facilitating the wall's structural organization (Bernard and Latgé, 2001; Bruneau et al., 2001).

In this study, we report the generation of mAb AP3 using *A. parasiticus* CWFs comprising a complex mixture of different antigens, and the characterization of its antigen specificity. The diversity, abundance and accessibility of potential antigens makes it challenging to identify the precise epitope (Schubert et al., 2018). The proteome and metabolome of *Aspergillus* spp. are highly dependent on the growth conditions, particularly stress-induced changes which are known to affect protein expression, cell wall composition, the production of secondary metabolites (Imanaka et al., 2010; Champer et al., 2016), and the abundance, chain length and composition of GM (Kudoh et al., 2015). The identification the epitope recognized by AP3 is also hampered by our use of different *Aspergillus* strains and cultivation conditions in different laboratory environments.

Despite the challenges described above, the specificity of mAb AP3 was confirmed in well-controlled replicate experiments using different *Aspergillus* strains and antigen compositions, leading to the successful localization and identification of the epitope. Indirect detection methods, such as ELISAs and deglycosylation assays indicated that AP3 detects substantial parts of a glycoantigen which is secreted into the culture medium, bound to the cell surface, and located on *Aspergillus* proteins. Importantly, immunofluorescence microscopy using the *Aspergillus* Δ*glfA* mutant strain, which is unable to synthesize Gal*f* residues (Schmalhorst et al., 2008), indicated that the epitope recognized by AP3 contains Gal*f* as a key constituent.

The *Aspergillus* proteins recognized by the antibody AP3 were identified by a combination of 2DE and mass spectrometry. Although we cannot be certain which of the proteins identified in the same spot was recognized by AP3, MS/MS analysis revealed that the antibody bound up to five distinct *Aspergillus* proteins that did not share any amino acid sequence similarity but carried multiple acceptor sites for *N*-linked and/or *O*-linked glycans, suggesting they are heavily glycosylated. The abundant glycosylation may explain the detection of these proteins in 2D gels at a higher molecular weights (>70 kDa) than expected. With the exception of Hsp70 and Hsp90, these glycoproteins carry a signal peptide causing them to be localized either in the cell wall (such as cell wall glucanase and amidase) or extracellular space (such as the mycelial catalase Cat1). Although Hsp90 is normally a cytosolic protein, it can travel to the fungal cell wall and regulate cell wall integrity (Lamoth et al., 2016). The lack of *N*-glycan acceptor sites and the very high number of potential *O*-glycosylation sites (122) in the cell wall glucanase, together with the strong fluorescence signal in the immunoblot (spots 13, 14, and 15) (Figure 4C), suggests that the AP3 antibody binds mainly to Gal*f* residues attached to *O*-linked glycan chains. Accordingly, the treatment of extracellular *Aspergillus* antigen and *A. flavus* CWPs with PNGase F did not affect the binding of mAb AP3, confirming that the signal was not associated with individual β-1,2-linked or α-1,2-linked Gal*f* residues attached to *N*-linked glycans and that AP3 binding was probably restricted to either β-1,5-linked or β-1,6-linked *O*-glycans and fungal-type GM (Komachi et al., 2013).

Finally, the direct glycoarray which uses 13 synthetic Gal*f* oligosaccharides resolved the linkage and length of the Gal*f* epitope detected by mAb AP3. These results provide a clear line of evidence that the Gal*f* pattern recognized by AP3 is characterized by a β-1,5-Gal*f* tetramer, whereas shorter oligosaccharides including a β-1,5-Gal*f* trimer are not detected. Interestingly, the replacement of the third β-1,5 Gal*f* unit with β-1,6 Gal*f* in the tetramer (oligosaccharide 12) destroys the antigen entirely. Therefore, the specificity of mAb AP3 is restricted to Gal*f*-containing structures in *Aspergillus* fungal-type GM and *O*-linked glycans ([β-D-Gal*f*-1,5]_4_), whereas *N*-glycans containing Gal*f* (α-1,2 Gal*f* ) and Gal*f*-containing glycosphingolipids (β-1,2 and or β-1,6 Gal*f* ) are not detected (Latge, 2009; Tefsen et al., 2012).

### Specificity of mAb AP3 Compared to Other Gal*f*-Specific Antibodies

Carbohydrates, such as GM and abundant immunodominant glycoproteins, often provide excellent biomarkers for fungal diseases because they are conserved among related species of fungi (Thornton and Wills, 2015). For example, *A. flavus* and *A. fumigatus* galactomannoproteins are recommended as biomarkers for the serological diagnosis of IA (Chan et al., 2002; Woo et al., 2003; Chong et al., 2004). Consequently, several GM-specific antibodies have already been developed for IA diagnostics, but thus far most of these antibodies belong to the IgM subclass (Thornton, 2010).

The IgM EB-A2 (Stynen et al., 1992) is the best-characterized Gal*f*-specific antibody used for the diagnosis of IA and has been regarded as the gold standard for more than 20 years. It is supplied as part of Bio-Rad's commercial Platelia *Aspergillus* EIA kit, which has been validated in several clinical studies and is approved by the FDA (Pfeiffer et al., 2006). Early epitope characterization studies suggested that EB-E2 recognized a tetramer of at least four β-1,5-linked Gal*f* moieties in *Aspergillus* GM, present in the cell wall and in glycoproteins (Stynen et al., 1992; Kudoh et al., 2015). However, a glycoarray was recently used to reinvestigate the oligosaccharide specificity of EB-A2, revealing that it also detects dimers and trimers with β-1,6 linkages (Krylov et al., 2019). This could explain the observed cross-reactivity between EB-A2 and non-*Aspergillus* fungi, contaminating GM in β-lactam antibiotics and foodstuffs, the cancer prodrug cyclophosphamide, and several other bacterial antigens, such as *Cryptococcus* galactoxylomannan (Dalle et al., 2005), which can generate false-positive results (Viscoli et al., 2004; Aubry et al., 2006; Zandijk et al., 2008).

Both EB-A2 and AP3 can also bind *Penicillium* spp., reflecting the presence of identical Gal*f* epitopes in these species (Unkefer and Gander, 1979). Cross-reactivity has also been reported between EB-A2 and *Fusarium* GM (Tortorano et al., 2012). Interestingly, we observed no cross-reactivity between AP3 and *F. oxysporum* Gal*f*-containing antigen preparations, which comprise β-1,6-linked β-D-Gal*f* residues with multiple side chains (Chen et al., 2015). This suggests that AP3 has a greater specificity for *Aspergillus* GM than EB-A2. AP3 was unable to detect lipoteichoic acid (LTA), a bacterial membrane polysaccharide substituted with β-1,5-linked D-Gal*f* residues (data not shown). We cannot exclude the possibility that AP3 binds to other fungi and bacteria, as well as cross-reacting antigens, such as LTA from *Bifidobacter* spp., but the specificity of AP3 for longer Gal*f* chains may reduce the likelihood of cross-reaction to other epitopes.

More recently, two novel Gal*f*-specific antibodies have been generated by immunizing mice with the synthetic pentasaccharide β-D-Gal*f*-1,5-[β-D-Gal*f*-1,5]_3_-α-D-Man*p*: mAb 7B8, which specifically recognizes the Gal*f* trimer, and mAb 8G4, which mainly detects the parental Gal*f* tetramer (Matveev et al., 2018). These are IgG antibodies like AP3 and they likewise detect defined Gal*f* epitopes located on the *Aspergillus* cell wall and glycoproteins as well as the secreted GM of several *Aspergillus* species. Neither 7B8 nor 8G4 react with *Bifidobacterium longum* and show less cross reactivity than EB-A2 (Mennink-Kersten et al., 2005). In contrast to AP3, 7B5, and 8G4 also recognize a shorter Gal*f* trimer ([β-D-Gal*f*-1,5]_3_) and a Gal*f-*dimer with a β-1,6 Gal*f* linkage (Matveev et al., 2018).

### Potential Applications of mAb AP3

In this study, we tested for the first time the potential of different Gal*f*-specific antibodies (EB-A2, L10-1, and AP3) to cooperate in the detection of Gal*f*-containing structures on AF-CWPs and *Aspergillus* GM. Interestingly, AP3 was unable to detect *Aspergillus* GM/EPS when used as the capture or detection reagent in a DAS-ELISA, whereas AF-CWPs were detected (data not shown). This is remarkable because detailed analysis of the EB-A2 antigen showed that GM contains more than 10 Gal*f* epitopes, making it possible to develop a DAS-ELISA with the GM-specific antibody acting as both the capture and detection antibodies (Stynen et al., 1992). AP3 can cooperate with L-10 and EB-A2 to detect AF-CWP and *Aspergillus* GM. However, *Aspergillus* GM could be not detected by an AP3 capture reagent with L10-1 as the detection antibody, although the reciprocal configuration was successful. The epitope detected by L10-1 has yet to be identified, so it is possible that L10-1 blocks the epitope detected by AP3 in this setup.

The glycosylation profiles of the proteins identified by 2DE were not analyzed in detail, but we speculate that the Gal*f*-epitope detected by AP3 is probably less abundant in secreted GM than CWPs, given that several such epitopes are accessible on the CWPs of *A. flavus*. The limited number of proteins specifically detected by AP3 in 2DE experiments, and the differential recognition of Gal*f*-containing epitopes by ELISA, suggest that the epitope is present on a limited number of Gal*f-*containing proteins and differs in this respect from the epitope recognized by L10-1 and EB-A2, thus making this antibody valuable for the detection of *Aspergillus* spp.

The unique ability of AP3 to bind Gal*f* oligosaccharides comprising four or more residues with β-1,5-linkages makes this antibody an ideal candidate for the detection of *Aspergillus* GM with higher sensitivity and specificity than current diagnostic reagents. It could also be used to monitor the distribution of long-chain Gal*f* oligosaccharides on fungal cell walls in combination with antibodies that recognize shorter oligosaccharide chains. Further studies are needed to validate the potential of AP3 and different Gal*f*-specific antibodies as a platform for the rapid analysis of galactofuranosylation and to distinguish between Gal*f*-containing structures on glycoproteins and fungal-type GM.

Compared to GM-specific and Gal*f*-specific IgMs, the greater stability and specificity of affinity-matured IgG antibodies, such as AP3, 7B8, and 8G4 may allow the development of novel detection assays for *Aspergillus* infections. Accordingly, the specificity of mAb AP3 for long-chain β-1,5 Gal*f* and its successful detection of GM in human serum demonstrates its value as a diagnostic tool. Although further evaluation of the AP3 sandwich assay is necessary with a larger number of patients, we have demonstrated the potential of AP3 for the rapid and sensitive diagnosis of IA.

In addition, the greater stability and reactivity of AP3 compared to IgM-based reagents could be advantageous in applications that involve the molecular imaging of *Aspergillus* infections *in vivo* (Rolle et al., 2016). The development of an IgG-based reagent could also provide therapeutic benefits (Di Mambro et al., 2019). For example, a β-glucan-specific IgG2b subtype antibody protected mice against infections with *Candida albicans*, whereas the corresponding IgM with an identical complementarity determining region did not (Torosantucci et al., 2009). The Gal*f*-specific IgM L10-1 did not confer a protective effect during an *A. fumigatus* infection (Heesemann et al., 2011). The recognition of *A. fumigatus* hyphae by the Fcγ receptor was shown to be necessary for opsonization (Gazendam et al., 2016). More recently, a humanized IgG targeting the *Crf* cell wall transglycosylase of *A. fumigatus* reduced the fungal burden in a neutropenic rat model (Chauvin et al., 2019). Therefore, mAb AP3 could also be developed as a therapeutic modality to recruit phagocytes to extracellular *Aspergillus* germ tubes, thus curing *Aspergillus* infections.

## Conclusion

AP3 is an IgG that recognizes *Aspergillus* Gal*f*-containing epitopes of four or more residues. It was generated by the immunization of mice with *A. parasiticus* CWFs and the subsequent production of hybridoma lines. The epitope detected by mAb AP3 is present in fungal-type GM and *O*-linked glycans on several *Aspergillus* glycoproteins. The carbohydrate specificity of AP3 was assessed using a thematic glycoarray comprising a series of synthetic oligosaccharide ligands structurally related to *Aspergillus* GM. Our data suggest that AP3 recognizes a β-1,5 Gal*f* tetramer that differs from the epitopes recognized by other GM/Gal*f*-specific mAbs. However, AP3 can also cooperate with other Gal*f*-specific mAbs to identify secreted GM and cell wall-associated glycoproteins in *Aspergillus* detection assays. In this context, AP3 could be developed as a valuable tool for *Aspergillus* cell wall and protein glycosylation studies, to screen crops for *Aspergillus* infection, and to diagnose IA with a lower risk of false positives.

## Data Availability

The raw data supporting the conclusions of this manuscript will be made available by the authors, without undue reservation, to any qualified researcher.

## Ethics Statement

All animal experiments were approved by the Landesamt für Natur, Umwelt und Verbraucherschutz Nordrhein-Westfalen (LANUV), reference number 8.87.-51.05.30.10.077. All animals received humane care according to the requirements of the German Tierschutzgesetz, §8 Abs. 1 and the Guide for the Care and Use of Laboratory Animals published by the National Institutes of Health.

## Author Contributions

MS, SS, GN, NN, and FE contributed to the conception and design of the study. MS, SX, AV, IC, VK, and NN performed the research and analyzed data. MS, SX, and AV helped with microscopy and ELISA experiments. IC and SX contributed with the 2DE experiments and mass spectrometry. VK and NN performed the glycoarray analysis. NN, FE, GN, and SS acquired funding. MS, SX, and NN prepared the original draft. All authors contributed to manuscript revision, read, and approved the submitted version.

### Conflict of Interest Statement

The authors declare that the research was conducted in the absence of any commercial or financial relationships that could be construed as a potential conflict of interest.

## Abbreviations

2DE: two-dimensional gel electrophoresis
ABPA: allergic bronchopulmonary aspergillosis
AF-CWP: *A. flavus* cell wall protein
CPA: chronic pulmonary aspergillosis
CWF: cell wall fragment
CWP: cell wall protein
DAS-ELISA: double antibody sandwich-ELISA
ELISA: enzyme-linked immunosorbent assay
Gal*f*: galactofuranose
GM: galactomannan
GPI: glycosylphosphatidylinositol
IA: invasive aspergillosis
Ig: immunoglobulin
mAb: monoclonal antibody
SD-Asp: *A. fumigatus* spent culture media.
