# Supplementary material for: Monoclonal Antibody AP3 Binds Galactomannan Antigens Displayed by the Pathogens Aspergillus flavus, A. fumigatus, and A. parasiticus
Source: Front Cell Infect Microbiol. 2019 Jul 16;9:234. doi: 10.3389/fcimb.2019.00234 (PMC6646516; doi:10.3389/fcimb.2019.00234)
Supplement: Supplementary file 1 [file Data_Sheet_1.docx]

Supplementary Material

## Supplementary Figures

**
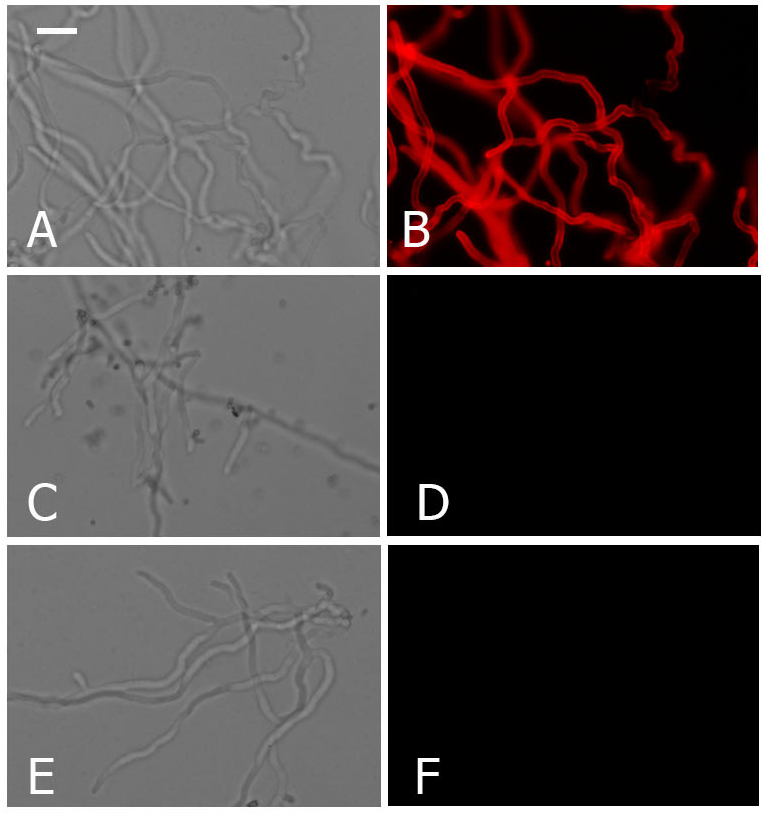
**

**Supplemental Figure 1**: Immunofluorescence analysis using mAb AP3 to detect *A. fumigatus* wild type and Δ*glfA* mutant strains. Mycelia of *A. fumigatus* (**A-B**) wild-type strain D141 and (**E-F**) mutant Δ*glfA* were immobilized on glass coverslips and detected with 200 µl AP3 hybridoma supernatant (25 µg/ml). For negative control mycelia of *A. fumigatus* (**C-D**) wild-type strain was incubated with PBS. Antibody binding was verified using secondary Cy3-labeled GAM IgM+IgG. For comparison, bright-field (**A, C, E**) and immunofluorescence images (**B, D, F**) are shown beside the corresponding immunofluorescence images. Scale bar = 20 µm.

**
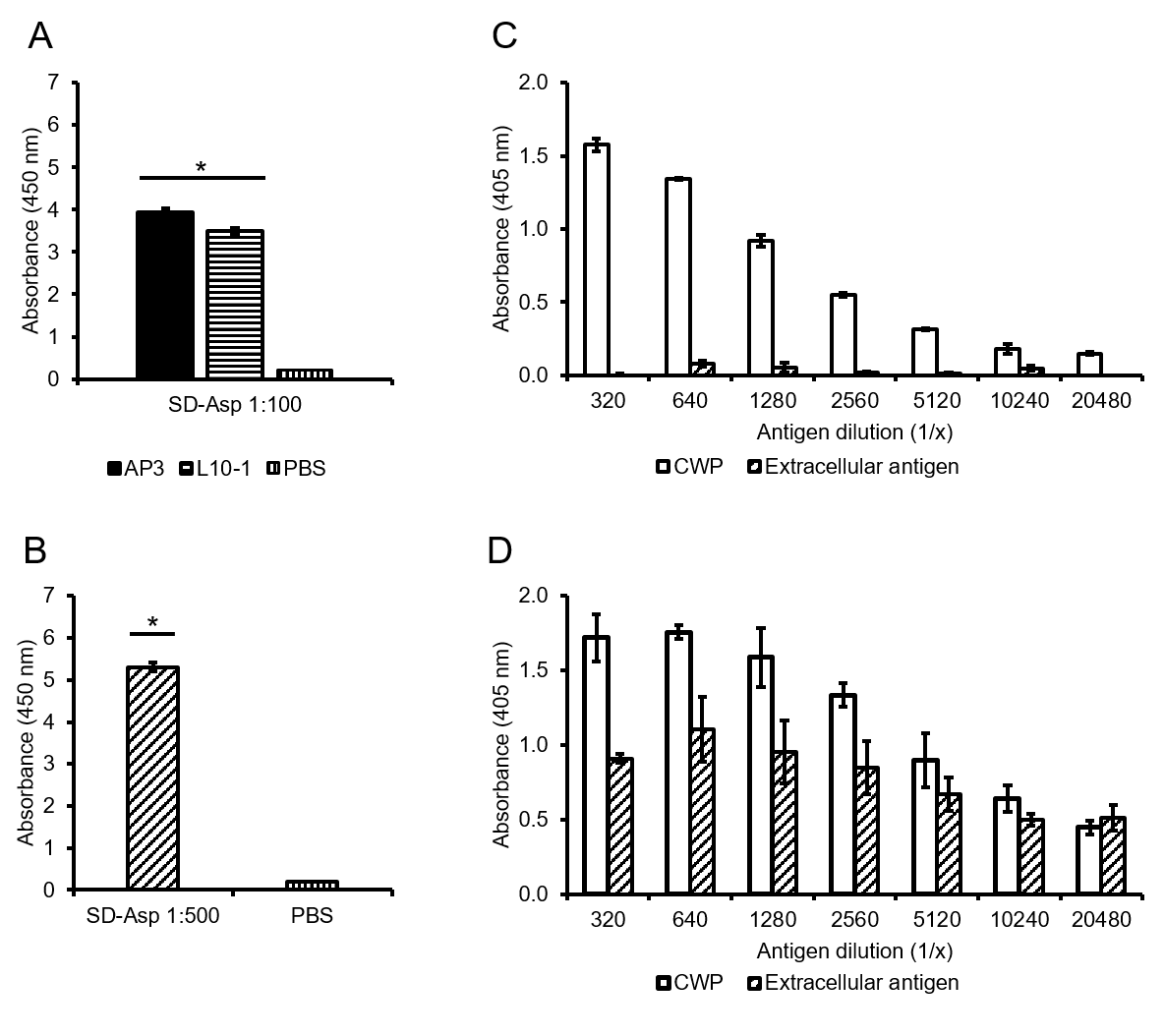
**

**Supplemental Figure 2**: Detection of *A. fumigatus* and *A. flavus* antigens by direct-coating ELISA and sandwich ELISA. (**A**) The GM standard SD-Asp (1:100) was directly coated onto microtiter plates. After blocking free binding sites with 3% (w/v) skimmed milk, GM was detected using purified mAb AP3 (400 ng/ml) or L10-1 (2 µg/ml) and secondary HRP-labeled goat-anti IgG or IgM specific antibodies. Absorbance was measured in triplicate after 20 min substrate incubation. (**B**) For the sandwich ELISA of *A. fumigatus* GM, SD-Asp was applied (1:500) onto microtiter plates coated with mAb AP3 (1:100) and bound GM was detected using L10-1 (2 µg/ml) followed by HRP-labeled goat anti-mouse IgM. For sandwich ELISA of *A. flavus* antigens, CWP and extracellular antigens (1:320–1:20,480) were applied onto microtiter plates coated with 900 ng/ml (**C**) L10-1 or (**D**) AP3. After blocking as described above, bound antigens were detected using 900 ng/ml AP3 (**C**) and L10‑1 (**D**) followed by HRP-labeled goat anti-mouse IgG (**C**) and goat anti-mouse IgM (**D**). Absorbance was measured in triplicate after 40 min substrate incubation. PBS: negative control. An asterisk denotes a statistically significant difference in antigen binding relative to the PBS negative control (*p*< 0.005).

**
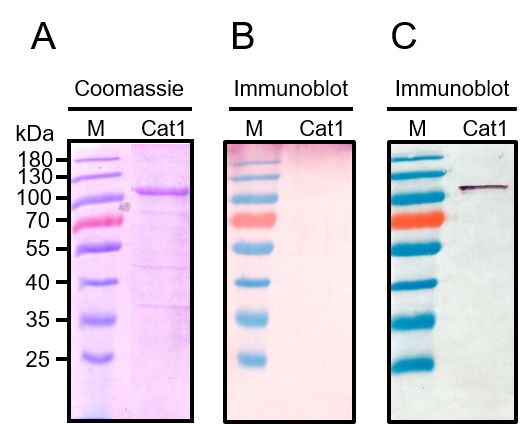
**

**Supplemental Figure 3**: Analysis of recombinant *A. flavus* mycelial catalase and detection by immunoblotting. *A. flavus* catalase was expressed in *E. coli* BL-21 and purified by Ni-NTA chromatography. Purified Cat1 was separated by SDS-PAGE and stained with (**A**) Coomassie Brilliant Blue or transferred to a nitrocellulose membrane (**B-C**). Immunoblot detection was carried out using 200 μl culture supernatant from monoclonal hybridoma cell line AP3 and GAM^AP^ H+L (120 ng/ml) (**B**) or rabbit anti-His_6_ and GAR^AP^ H+L (each 120 ng/ml) (**C**) followed by visualization using NBT/BCIP. M: Pre-stained protein marker (Fermentas), Cat1: Elution fraction of purified *A. flavus* mycelial catalase.

**
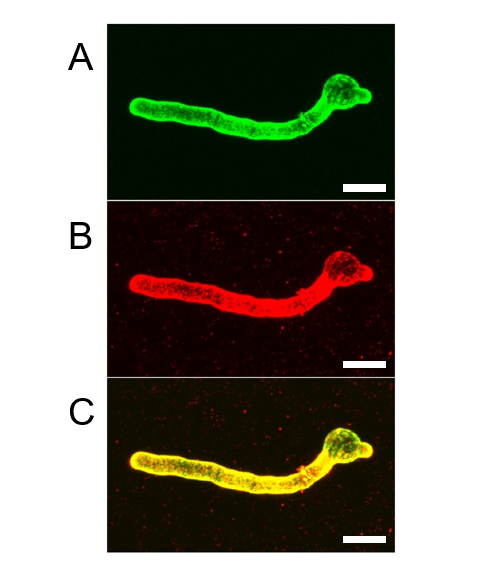
**

**Supplemental Figure 4**: Double labeling of *A. fumigatus* hyphae with mAb AP3 and L10-1. *Aspergillus* hyphae were fixed in 3.7% formaldehyde/PBS for 5 min, washed and incubated with Dylight 488-labeled (**A**) L10-1 and (**B**) purified AP3 followed by a secondary IgG antibody labeled with Dylight 550. Samples were analyzed using a Leica SP-5 confocal laser scanning microscope. (**C**) Overlay of A and B. Scale bar = 5 µm.

**
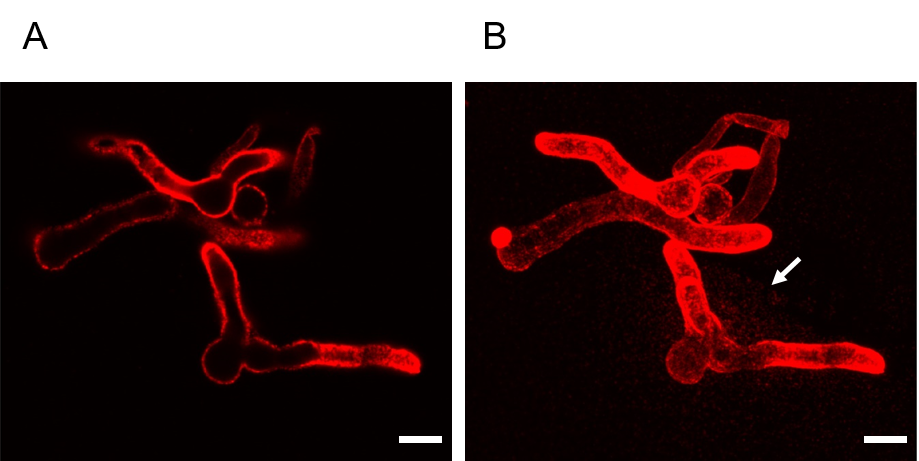
**

**Supplemental Figure 5**: Binding of mAb AP3 to *P. chrysogenum.*

Hyphae were immobilized on glass coverslips and dried overnight at 50°C. Samples were fixed in 3.7% formaldehyde/PBS for 5 min, washed and subsequently incubated with purified mAb AP3 and a secondary IgG antibody labeled with Dylight 550. Samples were analyzed using a Leica SP-5 confocal laser scanning microscope. Arrow indicates shedding of GM. (**A**) Confocal image, single plane. (**B**) Projection of all optical planes. Scale bar = 5 µm.
